# Structural Equation Model for Social Support and Quality of Life Among Individuals With Mental Health Disorders During the COVID-19 Pandemic

**DOI:** 10.2196/47239

**Published:** 2023-10-11

**Authors:** Bach Xuan Tran, Vu Anh Trong Dam, Pascal Auquier, Laurent Boyer, Guillaume Fond, Hung Manh Nguyen, Hung Tuan Nguyen, Huong Thi Le, Ha Nhi Thi Tran, Giang Thu Vu, Manh Duc Nguyen, Duong Anh Thi Nguyen, Bang Viet Ly, Carl A Latkin, Melvyn WB Zhang, Roger CM Ho, Cyrus SH Ho

**Affiliations:** 1 Institute for Preventive Medicine and Public Health Hanoi Medical University Hanoi Vietnam; 2 CEReSS, Research Centre on Health Services and Quality of Life Aix Marseille University Marseille France; 3 Mai Huong Daycare Psychiatric Hospital Hanoi Vietnam; 4 National Psychiatric Hospital No 1 Hanoi Vietnam; 5 Hanoi Department of Health Hanoi Vietnam; 6 School of Psychology The University of Queensland Brisbane Australia; 7 Bloomberg School of Public Health Johns Hopkins University Baltimore, MD United States; 8 Lee Kong Chian School of Medicine Nanyang Technological University Singapore Singapore Singapore; 9 Department of Psychological Medicine Yong Loo Lin School of Medicine National University of Singapore Singapore Singapore; 10 Institute for Health Innovation and Technology National University of Singapore Singapore Singapore

**Keywords:** contextual, social support, quality of life, mental health disorders, Vietnam, mental health, mental illness, cross-sectional, association

## Abstract

**Background:**

In light of the COVID-19 pandemic, the distribution of social support for mental health problems has likely become unequal. Family- and community-based social support has been recognized as a promising approach for mental disorders; however, limited global frameworks have been applied to developing countries such as Vietnam.

**Objective:**

The aim of this study was to evaluate the quality of life and social support among patients with mental health disorders in Vietnam and to investigate the factors associated with quality of life among these patients.

**Methods:**

A cross-sectional study was conducted on 222 psychiatric patients in Hanoi from 2020 to 2022. A structured questionnaire was developed based on four standardized scales: Mental Well-Being-5 scale, Multidimensional Scale of Perceived Social Support, EuroQoL-visual analog scale (EQ-VAS), and EuroQoL-5 dimensions-5 levels (EQ-5D-5L) scale. Tobit regression was used to identify factors associated with the EQ-5D-5L and EQ-VAS scores. Structural equation modeling was applied to verify the relationship between quality of life and social support.

**Results:**

The results showed that perceived support from family scored the highest compared to support from friends and significant others. Patients with depression reported the lowest quality of life and perceived social support. Structural equation modeling showed a root mean square error of approximation of 0.055 (90% CI 0.006-0.090), comparative fit index of 0.954, Tucker-Lewis index of 0.892, and standardized root mean squared error of 0.036 (*P*<.001). The hypothetical model indicated statistically significant correlations between EQ-VAS score and social support (*P*=.004), EQ-5D-5L and mental well-being (*P*<.001), and social support and mental well-being (*P*<.001). Critical deterioration of quality of life and inconsistency in social support for patients with mental illness were also recorded.

**Conclusions:**

There is a need to enhance social support and service delivery in Vietnam, focusing on occupation and quality of life. The correlations between social support, quality of life, and mental health issues suggest the potential of a clinical-social integrated intervention model of care.

## Introduction

Awareness of the burden posed by mental health disorders is steadily growing on a global scale, particularly in low- and middle-income countries [1]. According to a report by the World Health Organization (WHO), mental health disorders affected approximately 970 million people worldwide, constituting 12.8% of the population. These disorders are measured in terms of disability-adjusted life years (DALYs) and years lived with disability (YLDs), with mental health disorders accounting for over 40% of YLDs [2] and 15% of DALYs [3] globally. The prevalence of mental health disorders saw a sharp increase in 2020 due to the COVID-19 pandemic. Depressive symptoms were reported in 48.3% of the Chinese population, 32.7% of the Italian population, and 23.6% of the Spanish population [4]. In Vietnam, 45.8% of the population reported mild to moderate symptoms of stress following an extended period of social restrictions [5]. COVID-19 significantly impacted patients with pre-existing mental health disorders [6,7]. Strict social restrictions led to heightened stress levels and feelings of isolation among hospitalized patients [8]. Patients with mental illness were profoundly affected by changes in daily routines, social rhythms, and their sense of stability [9]. Additionally, socioeconomic factors such as remote working, wage cuts, and increased household expenditures were linked to the worsening of pre-existing disorders [10]. Given that mental health outcomes are influenced by a variety of biological, social, cultural, economic, and religious factors, it is crucial to approach mental health from a multidimensional perspective. The COVID-19 pandemic emphasizes the urgent need to shift the approach to mental health from a biomedical and therapeutic standpoint to a psychosocial perspective that maximizes family- and community-based interventions.

An increasing body of research has been dedicated to examining the influence of social determinants on health care outcomes. The WHO has officially acknowledged the significant impact of social factors on the prevalence and duration of mental disorders, attributing them to be responsible for shaping, sustaining, and enhancing overall health quality [11,12]. The UK National Health Service has emphasized the vital role of community connectors and advocated for an integrated approach that blends clinical treatment, mental health care, and social support [13]. Both frameworks acknowledge social support as a potent public health intervention, encompassing multitiered collaborations, including personal-interpersonal, personal-community, and interpersonal-community interactions. Furthermore, social support can function as a consistent source of care and a therapeutic approach for individuals with mental illnesses, without relying heavily on facility resources as is the case for many clinical interventions. Nevertheless, standardized models encounter the challenge of not fully capturing the influence of social support on diverse determinants of health, such as societal, cultural, and religious factors. Furthermore, since current frameworks are primarily rooted in Western contexts, there exists limited evidence regarding the effectiveness of the social support model in developing countries and resource-constrained settings [14-16].

As COVID-19 presented unique challenges to individuals with mental health disorders, it is critical to understand the role of social support in their quality of life (QoL). The standardized social support frameworks have been found to be limited in their applicability to the context of resource-scarce settings. While previous studies have identified unfavorable social outcomes for patients with mental health disorders, including social dysfunction, lack of social networks, severe stigma, and interpersonal challenges, contextualized social support models have neither been implemented nor studied for this population in Vietnam [17,18]. To contribute to the growing body of research on mental health care and social support in Vietnam, shedding light on the unique challenges faced by this population and identifying potential areas for intervention and improvement, we conducted this study with two main objectives: (1) to measure social support and QoL among patients with mental health disorders in Vietnam, and (2) develop and verify a structural model linking various factors that influence the QoL of patients with mental health disorders, particularly the social support aspect. Furthermore, we sought to confirm the bidirectional association between social support and QoL, where patients with mental health disorders suffer from low QoL, which in turn leads to a lack of social support; conversely, a lack of social support is identified as an underlying cause of mental disorders, resulting in lower QoL. Particularly in this time of COVID-19, where the pandemic has even further magnified the challenges faced by individuals with mental health disorders, such information can be invaluable in the development of tailored interventions that can strengthen social support and improve the QoL of patients.

**Figure 1:**
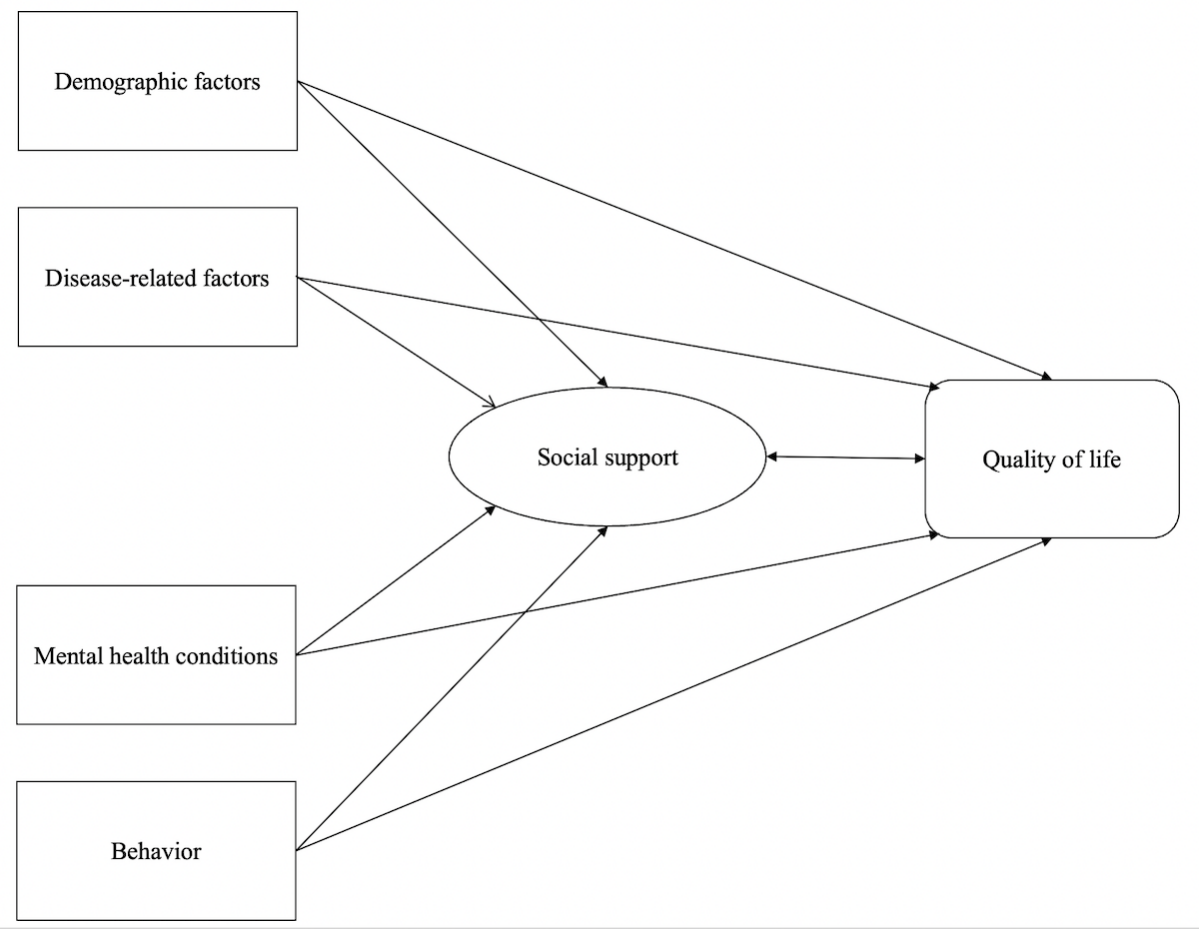
Conceptual framework of the study.

After reviewing the existing literature, we identified demographic, behavioral, illness-related, mental health–related, and social support factors that contribute to influencing the QoL of individuals with mental health conditions [19-21]. The conceptual framework employed in this study is presented in Figure 1.

## Methods

### Study Setting and Participants

From January 2020 to June 2022, a cross-sectional study was conducted in Hanoi, Vietnam. The eligibility criteria included patients who were 18 years old or above, currently being treated at the Mai Huong Daycare Psychiatric, willing to participate in the study through providing informed consent, and had the physical and mental capacity to answer the interviewer’s questions. Patients who had serious cognitive impairment or were unable to answer the data collectors’ questions were excluded from the recruitment process. A day psychiatric hospital model is currently being applied by Mai Huong Daycare Psychiatric, making the participants outpatients. The day-hospital model allows patients to spend less time at the hospital, reducing the potential negative effects of prolonged hospital stays, while facilitating faster recovery and improved QoL [22].

There were five investigators who were members of the research team and well-trained medical staff from Hanoi Medical University. The interview was conducted in a separate room where patient privacy was guaranteed. The patient was interviewed in a face-to-face manner using the questionnaire. The questionnaire required approximately 10-15 minutes to complete.

### Measure and Instruments

A structured questionnaire was used consisting of five main components: (1) demographic characteristics, (2) clinical history (chronic diseases, family psychiatric history, number of outpatient medical examinations, and number of mental health disorders examinations), (3) mental well-being (Mental Health Inventory-5 [MHI-5] scale), (4) social support (Multidimensional Scale of Perceived Social Support [MSPSS]), and (5) QoL (EuroQoL-5 dimensions-5 levels [EQ-5D-5L] scale and EuroQoL-visual analog scale [EQ-VAS]).

### Variables

#### Outcome Variables

The EQ-5D-5L scale was used to measure the QoL of participants through five domains: Mobility, Self-care, Usual Activities, Pain/Discomfort, and Anxiety/Depression. Each question had a score from 1 (“extreme problems”) to 5 (“no problems”), which resulted in 3125 possible health states from 1111 (worst health) to 55,555 (full health) [23]. One single “utility” score was defined for each health state and could be transformed using the interim scoring for EQ-5D-5L. This study used the validated Vietnamese version of the EQ-5D-5L with a score ranging from –0.5115 to 1 [24]. Higher scores indicated better QoL. The EQ-VAS is a vertical visual analog scale to self-assess health states from a score of 100 (best imaginable health) to 0 (worst imaginable health) [25].** **

The MSPSS was used with 12 items to measure the social support from the following subscales: family (4 items), friends (4 items), and significant others (4 items) [26]. Participants reported the MSPSS using a 7-point Likert scale ranging from 1 “strongly disagree” to 7 “strongly agree.” The score of subscales is the average score of the corresponding 4 items. Higher scores indicated higher support from family, friends, or significant others [26]. The maximum total score was 84, with a higher score indicating greater perceived social support. The MSPSS has been used widely for several groups such as those with infertility, people living with HIV/AIDS, and adolescents [27-29].

#### Covariates

##### Socioeconomic Status

Respondents were asked about their sociodemographic background, including age, gender, marital status, and occupation.

##### Substance Use Behaviors

We asked participants one question each about their current smoking and alcohol consumption behavior.

##### Clinical Characteristics

Participants were asked about their clinical characteristics, including comorbidities, chronic diseases, acute symptoms, family history of mood disorders, type of disorders (schizophrenia, bipolar disorder, depression, anxiety disorders, mixed anxiety-depressive disorder, sleep disorders, emotional disturbance, epilepsy, and other), family history of mental health disorders, the number of outpatient medical examinations, and the number of mental health disorders examinations.

The MHI-5 scale was used to measure the general mental health status of participants. There was a total of five items with each item scored from 1 to 5. The total score was measured by adding the total score of 5 questions and multiplying by 4. The total score ranged from 20 to 100 [30]. Higher scores indicated more severe mental health problems. The MHI-5 scale has been used for several groups such as adolescents and the general population [31,32].

### Sample Size and Sampling Method

Total population sampling was used in this study. The sample size was calculated using the formula to estimate the mean score of EQ-VAS among participants according to EQ-VAS scores reported from another study conducted in Vietnam [33]. This was selected for two main reasons. First, the study was conducted on adult patients quite similar to our study group. In Vietnam, there is still very little research on patients with mental illness. Second, the previous study used the same tool to measure the QoL of patients (EQ-VAS). Hence, we used α=.05, the mean score of EQ-VAS from the previous study of 66.3 (SD 12.5) [33], and a relative error of 0.025 to calculate the required sample size, resulting in a sample size of 219 patients. It was expected that 5% of those recruited would refuse to participate; therefore, we set the final sample size to N=230. At the end of data collection, 222 participants were recruited for the study.

### Statistical Analysis

The data analysis was conducted using Stata version 16 and missing data were managed using the listwise deletion method. Descriptive statistics are presented as the mean (SD) for continuous variables and frequencies and proportions for categorical variables. The Wilcoxon-Mann-Whitney test and Kruskal-Wallis test were used to test for differences in perceived social support and QoL scores across individual characteristics.

Individual characteristics, clinical history, mental well-being, and social support were considered potential covariates for the full models’ QoL. Multivariate Tobit regression was utilized to identify factors related to the EQ-VAS and EQ-5D-5L scores. Stepwise forward techniques were employed to create reduced models, with a significance level of *P*<.20 as the inclusion threshold. Statistical significance was set at *P*<.05. In contrast, the inferential statistics applied in this study utilized structural equation modeling, which involved employing maximum-likelihood estimation and path analysis to establish the implicit connection between the variables as originally hypothesized in Figure 1. Goodness-of-fit tests such as the goodness of fit index, comparative fit index (CFI), root mean square error approximation (RMSEA), Tucker-Lewis index (TLI), and standard root mean squared residual (SRMR) were used to evaluate the models.

### Ethical Considerations

This analysis used a part of the data set of a longitudinal study on individuals with and without mental health disorders in Vietnam, Vietnam’s Brain and Behavior Cohort, led by authors BXT and RCMH. The study monitors changes in functional near-infrared data and self-reported health outcomes using portable, functional near-infrared spectroscopy and clinical-behavioral assessments among Vietnamese patients, in an effort toward developing an artificial intelligence–based diagnosis system for psychiatric disorders in Vietnam.

The protocol of this study was approved by the Institutional Review Board of Hanoi Medical University (58/GCN-HĐĐNCYSH-ĐHYHN). We confirmed that all procedures were performed in accordance with relevant guidelines and regulations. Written informed consent was required from participants before participating in the study. Patients invited to participate were fully explained the content, purpose, and benefits of the study. The collected information was kept confidential and only used for research purposes. The data were encrypted to ensure confidentiality of the information.

## Results

Table 1 provides demographic information of the respondents, with a mean age of 43.1 (SD 13.4) years and a majority being men, single, having a high school education, and working as freelancers. The prevalence of chronic diseases was moderate (45%), with schizophrenia and bipolar affective disorder being the most commonly reported mental disorders. Participants reported an average of 8.9 outpatient medical examinations and 8.2 mental health disorder examinations in the past year.

**Table 1:** Individual characteristics of participants (N=222).

Characteristics		Value
**Gender, n (%)**		
	Man	122 (55.0)
	Woman	100 (45.0)
**Marital status, n (%)**		
	Single	106 (47.8)
	Married	96 (43.2)
	Divorced/separated/widow	20 (9.0)
**Education, n (%)**		
	Below high school	75 (33.8)
	High school	78 (35.1)
	University	69 (31.1)
**Occupation, n (%)**		
	Unemployed	50 (22.5)
	White-collar worker	18 (8.1)
	Blue-collar worker	22 (9.9)
	Student	28 (12.6)
	Retired	20 (9.0)
	Freelancer	84 (37.8)
**Chronic diseases, n (%)**		
	None	122 (55.0)
	One disease	62 (27.9)
	Two or more diseases	38 (17.1)
Family history of mental health disorders, n (%)		28 (12.7)
Drinking alcohol, n (%)		35 (15.8)
Smoking, n (%)		48 (21.6)
**Type of mental health disorder, n (%)**		
	Other	45 (20.5)
	Sleep disorders	27 (12.3)
	Schizophrenia	44 (20.0)
	Schizoaffective disorder	15 (6.8)
	Anxiety disorders	10 (4.6)
	Bipolar affective disorder	29 (13.2)
	Mixed anxiety disorder-depression	18 (8.2)
	Emotional disturbance	7 (3.2)
	Depression	14 (6.4)
	Psychosis	11 (5.0)
Age (years), mean (SD)		43.1 (15.4)
Number of outpatient medical examinations, mean (SD)		8.9 (11.6)
Number of mental health disorders examinations, mean (SD)		8.2 (10.9)

Table 2 presents the perceived social support scores, with mean scores of 5.08 (SD 1.09) for the family group, 4.32 (SD 1.52) for the friend group, 4.18 (SD 1.68) for significant others, and 54.33 (SD 13.52) for total perceived social support. Married participants reported higher scores for significant other support and total perceived social support than single participants (*P*=.03). Unemployed participants had the lowest perceived social support total score, while blue-collar workers had the highest perceived social support total score.

**Table 2:** Perceived social support regarding individual characteristics (N=222).

Characteristics			Family group support					Friend group support					Significant other support					Total perceived support		
			Mean (SD)		*P* value		Mean (SD)			*P* value		Mean (SD)			*P* value		Mean (SD)			*P* value
Total			5.08 (1.09)				4.32 (1.52)					4.18 (1.68)					54.33 (13.52)			
**Gender**					.64					.96					.45					.53
	Man	5.12 (1.09)				4.34 (1.52)					4.10 (1.70)					54.27 (13.74)				
	Woman	5.03 (1.10)				4.30 (1.52)					4.27 (1.67)					54.40 (13.31)				
**Marital status**					.06					.87					.03					.03
	Single	4.95 (1.23)				4.32 (1.63)					4.01 (1.70)					53.11 (14.76)				
	Married	5.28 (0.91)				4.33 (1.46)					4.47 (1.62)					56.32 (12.36)				
	Other	4.83 (1.01)				4.31 (1.27)					3.66 (1.72)					51.20 (10.96)				
**Education**					.47					.90					.53					.89
	Below high school	5.15 (1.08)				4.27 (1.53)					4.33 (1.66)					54.99 (12.76)				
	High school	5.00 (1.01)				4.35 (1.49)					4.10 (1.48)					53.77 (12.71)				
	University	5.10 (1.20)				4.36 (1.56)					4.11 (1.92)					54.25 (15.27)				
**Occupation**					.41					.28					.35					.47
	Unemployed	5.08 (1.21)				3.99 (1.75)					3.75 (1.82)					51.22 (15.59)				
	White-collar worker	5.29 (1.19)				4.51 (1.18)					4.06 (2.04)					55.44 (13.78)				
	Blue-collar worker	5.16 (1.10)				4.35 (1.83)					4.47 (1.78)					55.91 (15.86)				
	Student	4.65 (1.10)				4.32 (1.45)					4.03 (1.36)					52.00 (10.36)				
	Retired	5.19 (0.93)				3.88 (1.58)					4.43 (1.82)					53.95 (12.24)				
	Freelancer	5.13 (1.02)				4.58 (1.32)					4.38 (1.54)					56.39 (12.59)				
**Chronic diseases**					.27					.37					.44					.39
	None	5.18 (1.07)				4.38 (1.54)					4.26 (1.67)					55.25 (13.19)				
	One disease	4.85 (1.21)				4.22 (1.46)					3.94 (1.69)					52.08 (14.53)				
	Two or more diseases	5.14 (0.93)				4.32 (1.59)					4.31 (1.73)					55.05 (12.76)				
**Family history of mental health disorders**					.45					.69					.62					.65
	No	5.11 (1.10)				4.32 (1.55)					4.15 (1.72)					54.33 (13.50)				
	Yes	4.89 (1.09)				4.31 (1.39)					4.39 (1.49)					54.39 (14.12)				
**Drinking alcohol**					.16					.60					.43					.32
	No	5.12 (1.08)				4.34 (1.53)					4.21 (1.68)					54.64 (13.46)				
	Yes	4.89 (1.17)				4.25 (1.48)					4.04 (1.72)					52.69 (13.92)				
**Smoking**					.69					.50					.23					.34
	No	5.05 (1.12)				4.37 (1.48)					4.25 (1.65)					54.72 (13.33)				
	Yes	5.18 (1.01)				4.14 (1.67)					3.91 (1.79)					52.92 (14.22)				

Table 3 shows the QoL scores of the respondents, with mean scores of 67.99 (SD 20.51) for the EQ-VAS and 0.88 (SD 0.17) for EQ-5D-5L. Women had lower EQ-VAS scores than men (*P*=.01). Patients who were white-collar workers had the highest EQ-VAS and EQ-5D-5L scores, retired patients had the lowest EQ-VAS scores, and students had the lowest EQ-5D-5L scores. No significant differences were found between groups in EQ-5D-5L scores.

**Table 3:** Quality of life–related scores regarding individual characteristics (N=222).

Characteristics		EQ-VAS^a^			EQ-5D-5L^b^ index	
		Mean (SD)	*P* value		Mean (SD)	*P* value
Total		67.99 (20.51)			0.88 (0.17)	
**Gender**				.01		.09
	Man	71.80 (18.31)			0.89 (0.18)	
	Woman	63.34 (22.14)			0.86 (0.16)	
**Marital status**				.96		.79
	Single	68.08 (22.22)			0.88 (0.16)	
	Married	68.14 (18.75)			0.87 (0.20)	
	Other	66.75 (20.15)			0.88 (0.14)	
**Education**				.41		.47
	Below high school	66.13 (21.31)			0.86 (0.22)	
	High school	68.05 (18.09)			0.87 (0.16)	
	University	69.93 (22.26)			0.90 (0.12)	
**Occupation**				.01		.09
	Unemployed	67.04 (20.59)			0.86 (0.18)	
	White-collar worker	78.83 (15.88)			0.90 (0.14)	
	Blue-collar worker	67.41 (23.16)			0.86 (0.11)	
	Student	65.04 (24.49)			0.84 (0.19)	
	Retired	57.75 (17.51)			0.86 (0.14)	
	Freelancer	69.80 (18.98)			0.90 (0.19)	
**Chronic diseases**				.14		.32
	None	70.02 (20.30)			0.88 (0.20)	
	One disease	66.71 (21.67)			0.86 (0.16)	
	Two or more than two diseases	63.53 (18.83)			0.89 (0.11)	
**Family history of mental health disorders**				.76		.11
	No	68.02 (20.97)			0.88 (0.18)	
	Yes	68.04 (17.76)			0.87 (0.11)	
**Drinking alcohol**				.15		.31
	No	68.87 (20.44)			0.88 (0.17)	
	Yes	63.29 (20.58)			0.85 (0.16)	
**Smoking**				.51		.47
	No	67.26 (21.44)			0.87 (0.18)	
	Yes	70.60 (16.65)			0.89 (0.16)	

^a^EQ-VAS: EuroQoL-visual analog scale.

^b^EQ-5D-5L: EuroQoL-5 dimensions-5 levels.

Table 4 shows the characteristics of QoL and perceived social support based on the type of mental health disorder. Patients with psychosis had the highest EQ-5D-5L score, followed by schizophrenia and emotional disturbance (*P*=.03). Patients with sleep disorders had the highest total perceived social support score, followed by those with anxiety disorders (*P*=.01). In addition, patients with anxiety disorders had the highest family support scores (*P*=.06), whereas patients with sleep disorders had the highest support scores for the friend group (*P*=.04) and significant others group (*P*=.02).

**Table 4:** Characteristics of quality of life and perceived social support regarding the type of mental health disorders (N=222).

Mental health disorder	Quality of life, mean (SD)			Perceived social support, mean (SD)			
	EQ-VAS^a^	EQ-5D-5L^b^	Family group		Friend group	Other groups	Total
Total	67.90 (20.59)	0.88 (0.17)	5.07 (1.09)		4.31 (1.52)	4.16 (1.68)	54.18 (13.44)
Other	69.36 (20.07)	0.90 (0.12)	5.31 (1.04)		4.28 (1.56)	3.96 (1.87)	54.18 (10.16)
Sleep disorders	66.26 (21.79)	0.81 (0.30)	5.46 (0.88)		5.06 (1.03)	4.94 (0.86)	61.85 (9.45)
Schizophrenia	71.59 (18.94)	0.93 (0.10)	4.86 (0.97)		3.81 (1.58)	3.64 (1.62)	49.23 (13.97)
Schizoaffective disorder	65.67 (25.97)	0.80 (0.28)	5.18 (0.97)		4.07 (1.64)	4.00 (1.70)	53.00 (14.93)
Anxiety disorders	71.50 (17.00)	0.86 (0.13)	5.48 (0.89)		5.03 (1.42)	4.48 (1.96)	59.90 (14.88)
Bipolar affective disorder	68.07 (22.76)	0.86 (0.18)	4.80 (1.45)		4.40 (1.38)	4.54 (1.60)	54.97 (15.12)
Mixed anxiety disorder-depression	63.89 (18.44)	0.85 (0.13)	4.71 (1.04)		4.42 (1.60)	4.60 (1.78)	54.89 (14.00)
Emotional disturbance	72.71 (19.53)	0.91 (0.13)	4.86 (0.99)		4.93 (0.66)	4.39 (1.27)	56.71 (5.99)
Depression	57.14 (21.99)	0.83 (0.14)	4.77 (1.40)		3.50 (1.60)	3.14 (1.86)	45.64 (15.28)
Psychosis	67.73 (18.35)	0.96 (0.07)	5.32 (0.90)		4.48 (1.65)	4.59 (1.40)	57.55 (14.11)

^a^EQ-VAS: EuroQoL-visual analog scale.

^b^EQ-5D-5L: EuroQoL-5 dimensions-5 levels.

Table 5 displays the factors related to the QoL of participants. Older age, being a woman, being married, and drinking alcohol were negative factors that decreased the QoL score. Greater mental well-being was a positive factor that increased both the EQ-VAS and EQ-5D-5L scores. Patients who had higher perceived social support had a higher EQ-VAS score. The full multivariate tobit regression models to identify factors associated with the QoL of participants are presented in Multimedia Appendix 1.

**Table 5:** Factors related to the quality of life of participants (N=222).

Factors				EQ-VAS^a^ score, coefficient (95% CI)		EQ-5D-5L^b^ index, coefficient (95% CI)
**Socioeconomic factors**						
	Age (years)		–0.23 (–0.39 to –0.06)		N/A^c^	
	Gender (woman vs man=reference)		–10.00 (–15.10 to –4.90)		N/A	
	**Marital status (vs single=reference)**					
		Married	N/A		–0.10 (–0.17 to –0.03)	
		Other	N/A		0.04 (–0.08 to 0.16)	
Number of outpatient medical examinations (times)				–0.50 (–1.01 to 0.01)		N/A
Number of mental health disorders examinations (times)				0.41 (–0.13 to 0.96)		N/A
Drinking alcohol (yes vs no=reference)				–7.57 (–14.51 to –0.63)		N/A
**Mental health**						
	MHI-5^d^ score		0.34 (0.23 to 0.45)		0.005 (0.004 to 0.007)	
	Perceived social support score		0.31 (0.11 to 0.50)		N/A	

^a^EQ-VAS: EuroQoL-visual analog scale.

^b^EQ-5D-5L: EuroQoL-5 dimensions-5 levels.

^c^N/A: not applicable.

^d^MHI-5: Mental Health Inventory-5.

**Figure 2:**
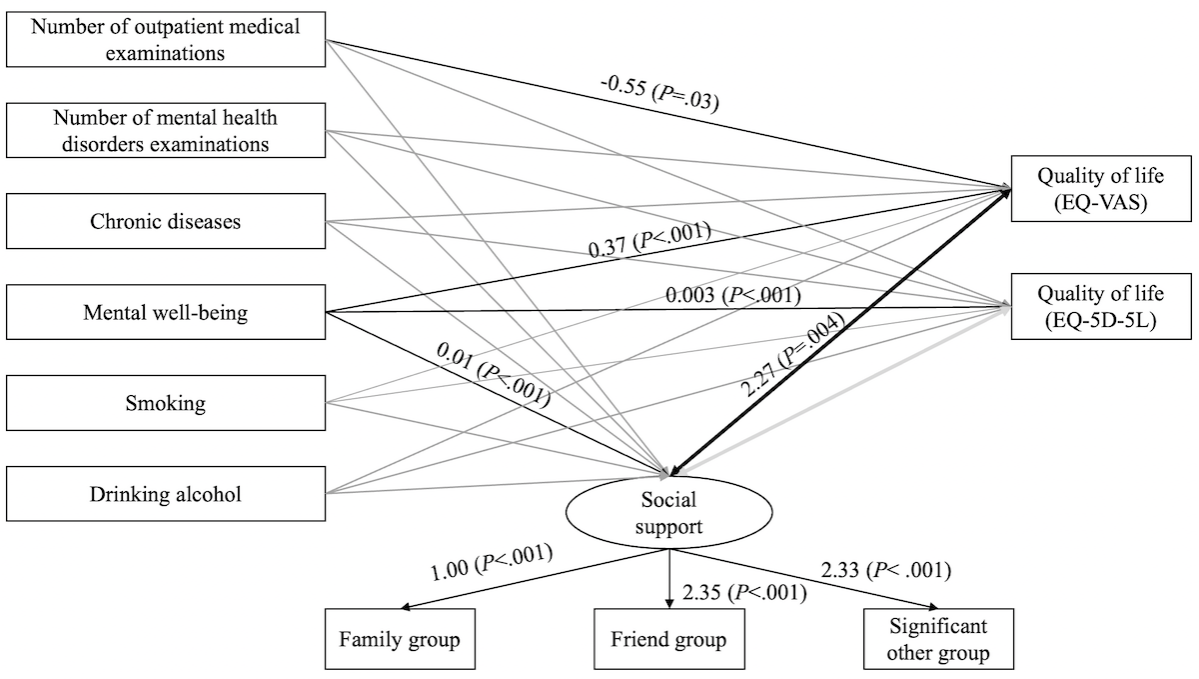
Structural model and standardized path coefficients (N=222). EQ-5D-5L: EuroQol-5 dimensions-5 levels; EQ-VAS: EuroQoL-visual analog scale.

Figure 2 presents the results of the structural model and standardized path coefficients of the hypothetical model. The analysis of the structural equation model using the study variables in the hypothetical model resulted in an RMSEA of 0.055 (90% CI 0.006-0.090), CFI of 0.954, TLI of 0.892, and SRMR of 0.036 (*P*<.001). The structural equation model showed that mental well-being was significantly related (*P*<.001) to the QoL score (coefficient=0.37, 95% CI 0.27-0.47 for EQ-VAS; coefficient=0.003, 95% CI 0.002-0.004 for EQ-5D-5L) and with social support (coefficient=0.01, 95% CI 0.004-0.013). Additionally, the model revealed a bidirectional relationship between social support and EQ-VAS (coefficient=2.27, 95% CI 0.74-3.81; *P*=.004). Table 6 illustrates the full models, including the outcome and both tier-1 and tier-2 predictors.

**Table 6:** Full models, including the outcome and both tier-1 and tier-2 predictors.

Structural component		Standardized path coefficient	*P* value of unstandardized estimate
**Direct paths: tier 2 to tier 1**			
	Number of outpatient medical examinations to Social support	–0.014	.11
	Number of mental health disorders examination to Social support	0.011	.21
	Chronic diseases to Social support	–0.013	.80
	Mental well-being to Social support	0.009	<.001
	Smoking to Social support	–0.136	.18
	Drinking alcohol to Social support	0.056	.63
**Direct paths: tier 2 to the outcome**			
	Number of outpatient medical examinations to EQ-VAS^a^	–0.554	.03
	Number of mental health disorders examination to EQ-VAS	0.413	.14
	Chronic diseases to EQ-VAS	–3.172	.05
	Mental well-being to EQ-VAS	0.371	<.001
	Smoking to EQ-VAS	4.312	.18
	Drinking alcohol to EQ-VAS	–5.620	.12
	Number of outpatient medical examinations to EQ-5D-5L^b^	–0.002	.50
	Number of mental health disorders examinations to EQ-5D-5L	0.001	.59
	Chronic diseases to EQ-5D-5L	0.002	.91
	Mental well-being to EQ-5D-5L	0.003	<.001
	Smoking to EQ-5D-5L	0.018	.52
	Drinking alcohol to EQ-5D-5L	–0.022	.50
**Measurements**			
	Social support to family group	1.000	Constrained
	Social support to friend group	2.352	<.001
	Social support to significant others groups	3.056	<.001
**Bidirectional relationships**			
	Social support and EQ-VAS	2.274	.004
	Social support and EQ-5D-5L	–0.001	.90

^a^EQ-VAS: EuroQoL-visual analog scale.

^b^EQ-5D-5L: EuroQoL-5 dimensions-5 levels.

## Discussion

### Principal Results

This study revealed a decline in QoL and unequal distribution of social support among patients with mental health disorders, as students experienced the lowest social support and workers perceived the highest levels of social support. Depression, sleeping disorders, and bipolar-related problems were the most significant mental health concerns recorded. The current resource allocation scheme was problematic, as all common mental health issues had critically lower levels of social support than other disorders. This study found that QoL decreased with age, among women, and for those with alcohol consumption. The most prevalent source of support across all patient groups was family support, followed by friend groups, implying a potential design of integrated community- and family-based interventions for mental health issues in resource-scarce settings.

### Comparison With Prior Work

This study did not measure any COVID-19–related variables; however, since the study was conducted during the severe COVID-19 epidemic, the results can provide a comprehensive picture of mental health disorders in Vietnam during COVID-19. Overall, the QoL among patients with mental health disorders was lower than that of the general Vietnamese population [34]. Patients with mental health disorders experience impairments in daily tasks; isolation; and a decrease in self-sufficiency, self-confidence, and self-esteem.

In this study, participants only received outpatient treatments. Therefore, in addition to attending medical appointments, many patients still work and carry out daily tasks. Our study revealed that patients with mental health disorders had the lowest scores related to perceived support from friend groups. Amal et al [35] conducted a study on psychiatric patients in Egypt and reported similar findings. This finding is likely related, in part, to stigma and prejudice, which have a direct impact on the social opportunities for individuals with mental illnesses. Furthermore, the general population is unaware of the consequences of mental illness and many are often terrified by people with these health conditions. By contrast, this study revealed that the patients had the highest social support from family. This result can be explained by the fact that since our participants were outpatients, they spent most of their time with their family members. Family relationships are strong in Southeast Asia, which may be beneficial if they are used as social support rather than social coercion. Many patients with major mental illnesses either live with their families (parents, spouses, siblings, and children) or have regular contact with them. Goldberg et al [36] evaluated the impact of social networks among individuals with mental impairments in the United States, and discovered that the closest relatives were the most commonly utilized source of support. Furthermore, Brunt and Hansson [37] discovered that patients in in-patient and assisted community settings in Sweden had a large proportion of family members in their social networks.

Our study revealed inconsistent patterns in QoL and support availability among different occupations and mental disorders. Students experienced the lowest social support and EQ-5D-5L scores. Blue-collar workers received the highest levels of social support but experienced the lowest QoL compared to other occupation groups. Understandably, stable employment tends to provide certain benefits such as secure incomes, health insurance (though not yet covering mental disorders), and support systems [38]. The work characteristics of many blue-collar workers in Vietnam and other countries involve intensive and heavy manual labor tasks and appalling working environments, which could lead to a low QoL, even in the usual context that is amplified in the presence of mental disorders. Likewise, for treated and returning-to-work patients, sudden re-exposure to hectic work routines could also lead to loss of job satisfaction or even to relapse of previous conditions [39]. This study highlights the urgent need to develop workplace-based management standard practices that support and distribute the workload to help address the health status of treated and returning-to-work patients.

According to several studies, depression, a mix of anxiety-depression, and sleeping disorders are common determinants of low QoL, not only among mental health patients but across all population groups [40-42]. Patients with these conditions have been found to have the lowest QoL [40-42]. However, our study found that the level of social support provided for depression in particular was critically lower than that for other illnesses. This finding suggests that the distribution of social support is not linked to the needs of patients with mental health disorders [43]. The social support system focuses on illness severity as an indicator rather than on QoL. Although the impairment caused by most cases of depression, anxiety, and sleeping disorders is generally not as severe as that associated with other mental health conditions such as schizophrenia or psychosis, the symptomology of conditions is constantly present in daily life, hardly eradicated, and often only treated with outpatient care [43]. This means that patients diagnosed with these conditions have to bear the burden of their illnesses while maintaining normal social functioning such as working, caregiving, or housekeeping. As a result, patients with mental health disorders are subject to induced stress and feelings of inadequacy or become overwhelmed when their responsibilities are not met, which amplifies their mental health disorders and can create a vicious cycle that significantly impacts their holistic well-being. Instead of relying on illness severity to allocate social support resources, health care professionals should focus on QoL as an indicator for more effective distribution. Moreover, patients may deliberately avoid social support due to insecurity about their health conditions and fear of the social stigma associated with such conditions [44,45]. Interian et al [46] suggested that social stigma not only prohibits patients from seeking social support but also from seeking care altogether. Therefore, additional studies should be conducted on the correlation between social stigma and social support among patients with mental health conditions to better understand and promote help-seeking behavior among this vulnerable population.

Patients with mental disorders have lower QoL and lack social support. The structural equation model analysis confirmed the bidirectional relationship between social support and EQ-VAS scores. Previous research has linked the lack of social support to social isolation, loneliness, and higher risks of physical health problems [47], which can lead to the development of mental health issues. However, various studies, such as the meta-analysis performed by Harandi et al [48] or the cross-sectional study by Connell et al [49] have confirmed the relationships between social support, QoL, and mental health disorders, but only separately. The present study suggests that interventions to improve each of these domains can be achieved through the two other domains, which are rarely included in the literature. For example, strengthening family support has major impacts on reducing mental health symptoms immediately and in the long term [50,51], while social support availability and QoL should be integrated into the screening standard for early diagnosis and prevention. A longitudinal study conducted over the course of 23 years with 1-year, 4-year, 10-year, and 23-year follow-ups indicated the sustainability of family members as a source of support during treatment and recovery [52]. However, further clinical studies are needed to determine the extent to which social support and QoL can serve as indicators for diagnoses of mental health problems. Given the bidirectional association between social support and QoL and the impacts of their interplay on mental health issues, efforts should be directed to both ends to optimize treatment outcomes and prevent long-term impacts on well-being [52].

### Implications

This study offers several implications for mental health treatment and interventions. First, given the outpatient nature of treatment where patients continue to work and carry out daily tasks alongside their therapy appointments, and considering the long-term and often lifelong nature of mental health treatment, it is crucial to provide stable and lifelong sources of social support such as family-based support, which has been shown to have a substantial impact on treatment outcomes [53,54]. Family-based interventions should be standardized to suit the Vietnamese context, based on successes and challenges in countries that have adopted social support using prior standardized frameworks [14,55,56]. Guidelines for family engagement should be provided for the care and service delivery of patients with mental health conditions. Additionally, constant feedback and follow-ups between clinicians and patients are essential to optimize treatment quality and measure progress. Second, a model of “work therapy” should be implemented to provide treatments as work tasks in the workplace, especially for labor workers who experience low levels of social support, to prevent detachment from the working routine and help patients quickly readapt to work after treatment. Finally, mental health treatment should be delivered through a combination of approaches, including social support, psychological therapy, family-based interventions, and work-related treatments.

### Strengths and Limitations

The main strength of this study is that the majority of participants only received outpatient treatments, allowing for the investigation of variables in general settings such as daily life and the workplace.

However, our study had several limitations. First, we were unable to analyze causal relationships due to the cross-sectional study design. As a result, we cannot be certain whether the capacity of the patients was impacted by social support or other reasons. Second, self-reported data may suffer from a limitation of recall bias, and social desirability bias may cause certain answers to be underestimated or overestimated. Furthermore, because the time of data collection varied, recall bias might be a concern that leads to inaccuracies, especially for patients with mental health disorders. The population sampling technique may have limited the generalizability of the results for Vietnamese patients. Finally, our study was conducted during the COVID-19 pandemic, limiting the generalizability of the study results in a postpandemic period. Despite these limitations, the study identified significant determinants and trends, enabling a proposal of multilevel interventions for mental health issues and social support.

### Conclusions

Our study sheds light on the challenges faced by individuals with mental health disorders in Vietnam, including a decrease in QoL and barriers to accessing social support. The QoL model was used to fit a structural equation model to systematically verify and analyze the relationship between QoL and other variables with social support as a mediator. We found that mental well-being, QoL, and social support are interconnected in a complementary manner, suggesting that improving one factor can positively impact the others. However, further research is needed to generalize these findings to other populations before implementing a standardized framework. Our study identified several issues within the current social support system, including inadequate support for labor workers with mental health issues and a severity-based approach to resource allocation rather than one that prioritizes QoL. Finally, our study highlights the need for a shift in the mental health intervention approach, and we propose an integrated model of family-based and therapeutic interventions to address mental health disorders.

## Appendix

Multimedia Appendix 1 Full multivariate tobit regression models to identify factors associated with the quality of life of participants (N=222).

## Abbreviations

CFI: comparative fit index
DALYs: disability-adjusted life years
EQ-5D-5L: EuroQoL-5 dimensions-5 levels
EQ-VAS: EuroQoL-visual analog scale
MHI-5: Mental Health Inventory-5
MSPSS: Multidimensional Scale of Perceived Social Support
QoL: quality of life
RMSEA: root mean square error approximation
SRMR: standard root mean squared residual
TLI: Tucker-Lewis index
WHO: World Health Organization
YLDs: years lived with disability

## Data Availability

All data generated or analyzed during this study are included in this published article.
