# Supplementary material for: Structural Equation Model for Social Support and Quality of Life Among Individuals With Mental Health Disorders During the COVID-19 Pandemic
Source: JMIR Public Health Surveill. 2023 Oct 11;9:e47239. doi: 10.2196/47239 (PMC10600649; doi:10.2196/47239)
Supplement: Multimedia Appendix 1 [file publichealth_v9i1e47239_app1.docx]

**Multimedia Appendix 1**

**Full multivariate tobit regression models to identify factors associated with the quality of life of participants (N=222)**

| **Factors** | **EQ-VAS score** | | **EQ-5D-5L Index** | |
| --- | --- | --- | --- | --- |
|  | **Coef** | **95%CI** | **Coef** | **95%CI** |
| **Socio-economic** |  |  |  |  |
| **Age** *(unit: year)* | -0.07 | -0.34; 0.20 | -0.0004 | -0.004; 0.004 |
| **Gender** *(Female vs Male - Ref)* | -10.12 ^a^ | -15.98; -4.25 | -0.01 | -0.09; 0.07 |
| **Marital status** *(vs Single - Ref)* |  |  |  |  |
| Married | -2.15 | -8.66; 4.35 | -0.10 ^b^ | -0.19; -0.01 |
| Other | 6.53 | -2.96; 16.03 | 0.03 | -0.10; 0.17 |
| **Education** *(vs Below high school education - Ref)* |  |  |  |  |
| High school education | 3.23 | -2.73; 9.20 | 0.02 | -0.06; 0.10 |
| Higher school education | 2.95 | -3.79; 9.69 | 0.04 | -0.05; 0.14 |
| **Occupation** *(vs Unemployed - Ref)* | |  |  |  |
| White-collar worker | 8.59 | -2.46; 19.64 | -0.01 | -0.16; 0.14 |
| Blue-collar worker | 3.41 | -5.86; 12.68 | -0.02 | -0.14; 0.11 |
| Student | 2.86 | -7.57; 13.29 | -0.02 | -0.17; 0.12 |
| Retire | -4.73 | -15.21; 5.75 | 0.03 | -0.12; 0.17 |
| Freelancer | -0.70 | -7.27; 5.88 | 0.02 | -0.07; 0.11 |
| **The number of outpatient medical examination** *(unit: time)* | -0.47 | -0.99; 0.05 | -0.003 | -0.01; 0.004 |
| **The number of mental health disorders examination** *(unit: time)* | 0.40 | -0.15; 0.96 | 0.002 | -0.01; 0.01 |
| **Family history of mental health disorders** *(Yes vs No - Ref)* | 0.32 | -6.94; 7.59 | -0.04 | -0.14; 0.06 |
| **Chronic diseases** *(vs None - Ref)* |  |  |  |  |
| One disease | -0.09 | -5.92; 5.73 | -0.02 | -0.10; 0.06 |
| Two or more than two diseases | -2.33 | -10.05; 5.39 | 0.01 | -0.10; 0.11 |
| **Drinking alcohol** *(Yes vs No - Ref)* | -8.66 ^b^ | -16.04; -1.27 | -0.03 | -0.14; 0.07 |
| **Smoking** *(Yes vs No - Ref)* | 2.24 | -5.07; 9.55 | 0.03 | -0.07; 0.13 |
| **Mental health** |  |  |  |  |
| MHI-5 score *(unit:score)* | 0.34 ^a^ | 0.23; 0.46 | 0.005 ^a^ | 0.003; 0.01 |
| **Social support** |  |  |  |  |
| Perceived Social Support score *(unit:score)* | 0.34 ^a^ | 0.15; 0.54 | 0.001 | -0.002; 0.003 |
| ^a^*P*<.01, ^b^*P*<.05 |  |  |  |  |
